# Supplementary material for: High-Throughput Morphological Profiling of Patient-Derived Human Liver Organoids for Hepatotoxicity Hazard Identification and Compound Prioritization
Source: bioRxiv. 2026 Oct 4:2026.09.29.755355. Preprint. [Version 1] doi: 10.64898/2026.09.29.755355 (PMC13648574; doi:10.64898/2026.09.29.755355)
Supplement: Supplementary file 1 [file NIHPP2026.09.29.755355V1-supplement-1.pdf]

bioRxiv preprint doi: https://doi.org/10.64898/2026.09.29.755355; this version posted October 4, 2026. The copyright holder for this preprint (which was not certified by peer review) is the author/funder, who has granted bioRxiv a license to display the preprint in perpetuity. It is made available under a CC-BY 4.0 International license.

Supplemental Figures

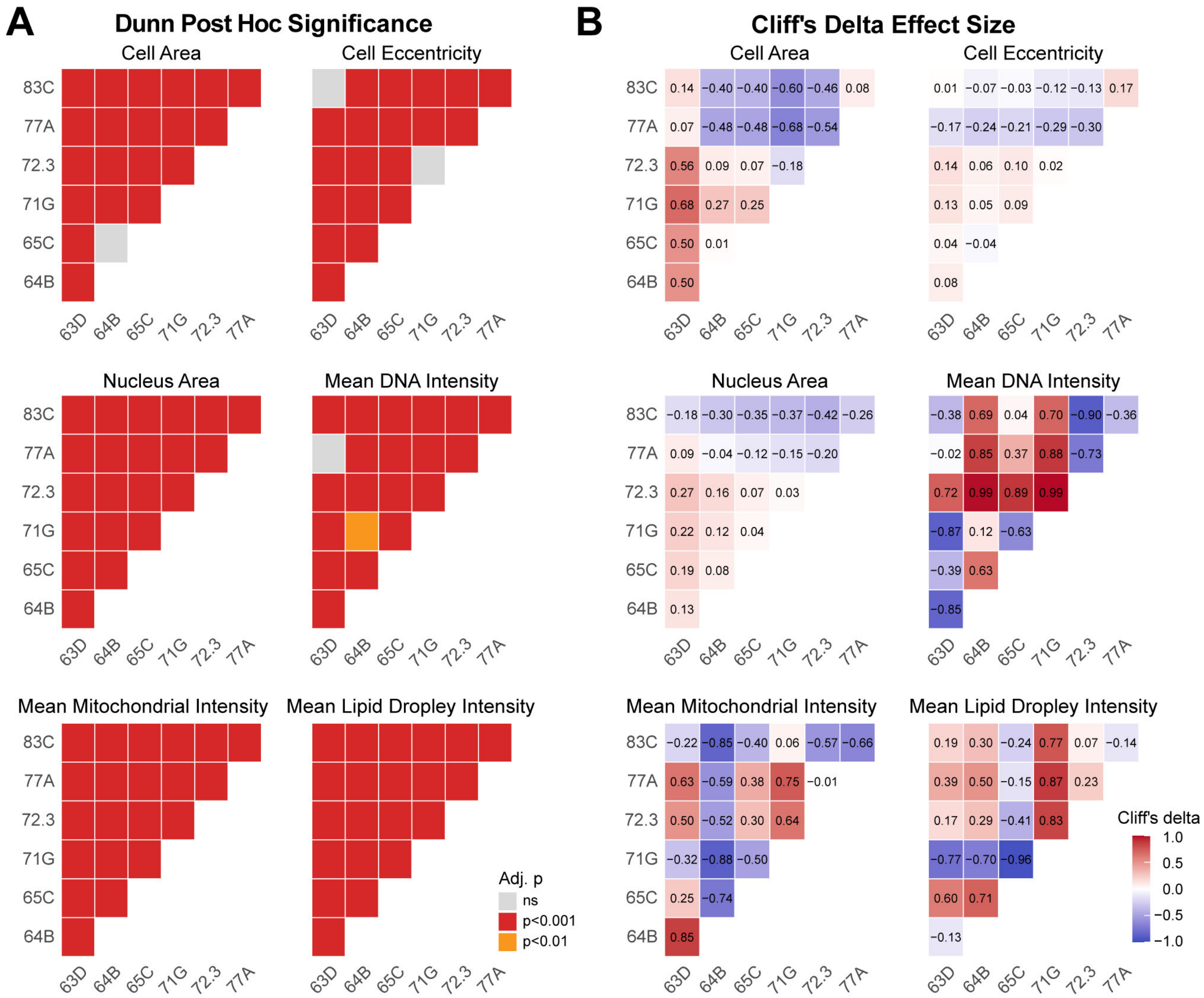

Supplemental Figure S1. Pairwise statistical comparisons of baseline cellular features across HLO models.

(A) Heatmaps showing pairwise differences among HLO models for representative cellular features. Statistical significance was assessed using Kruskal–Wallis testing followed by Dunn's post hoc multiple-comparison analysis with Benjamini–Hochberg adjusted *p* values. Gray indicates non-significant differences, whereas warmer colors indicate increasing statistical significance. **(B)** Heatmaps of Cliff's delta effect sizes for the same pairwise comparisons. Cliff's delta quantifies the magnitude and direction of differences between groups. Red indicates higher values in group 1 relative to group 2, blue indicates lower values, and white indicates similar distributions. Values within tiles denote Cliff's delta estimates, highlighting donor-dependent baseline phenotypic differences across HLO models.

bioRxiv preprint doi: https://doi.org/10.64898/2026.09.29.755355; this version posted October 4, 2026. The copyright holder for this preprint (which was not certified by peer review) is the author/funder, who has granted bioRxiv a license to display the preprint in perpetuity. It is made available under a CC-BY 4.0 International license.

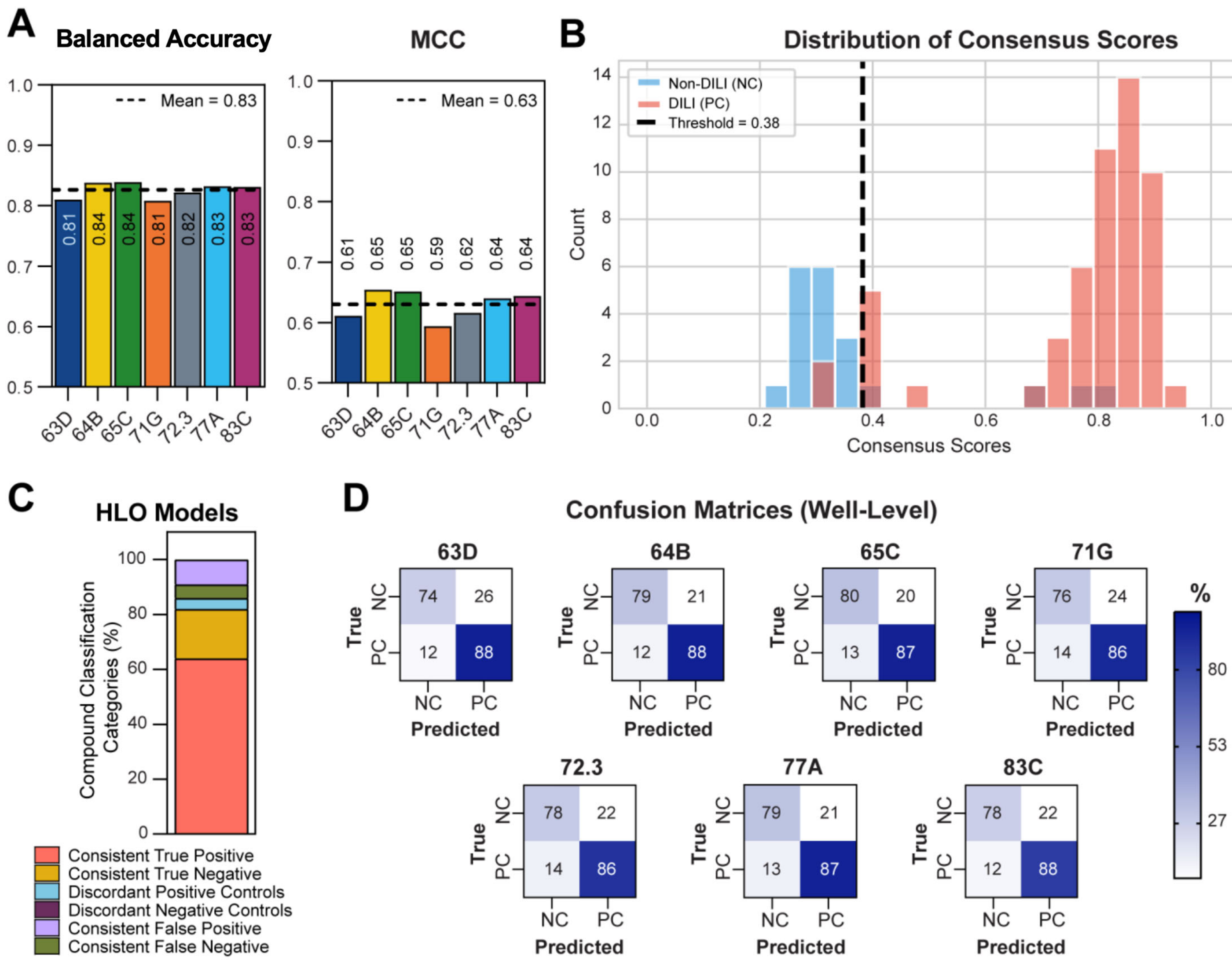

Supplemental Figure S2. Well-level performance and classification metrics across individual and consensus HLO models. A) Balanced accuracy (left) and MCC (right) for each HLO model. These metrics demonstrate consistent predictive performance across genetically diverse donors. **(B)** Distribution of consensus DILI scores at the well level, showing separation between non-hepatotoxic (negative) and hepatotoxic (positive) wells. **(C)** Well-level compound classification categories for each HLO model and the consensus model, indicating the proportion of correctly classified hepatotoxic and non-hepatotoxic wells. **(D)** Confusion matrices calculated at the well level for all seven HLO models, highlighting sensitivity, specificity, and overall accuracy.

bioRxiv preprint doi: https://doi.org/10.64898/2026.09.29.755355; this version posted October 4, 2026. The copyright holder for this preprint (which was not certified by peer review) is the author/funder, who has granted bioRxiv a license to display the preprint in perpetuity. It is made available under a CC-BY 4.0 International license.

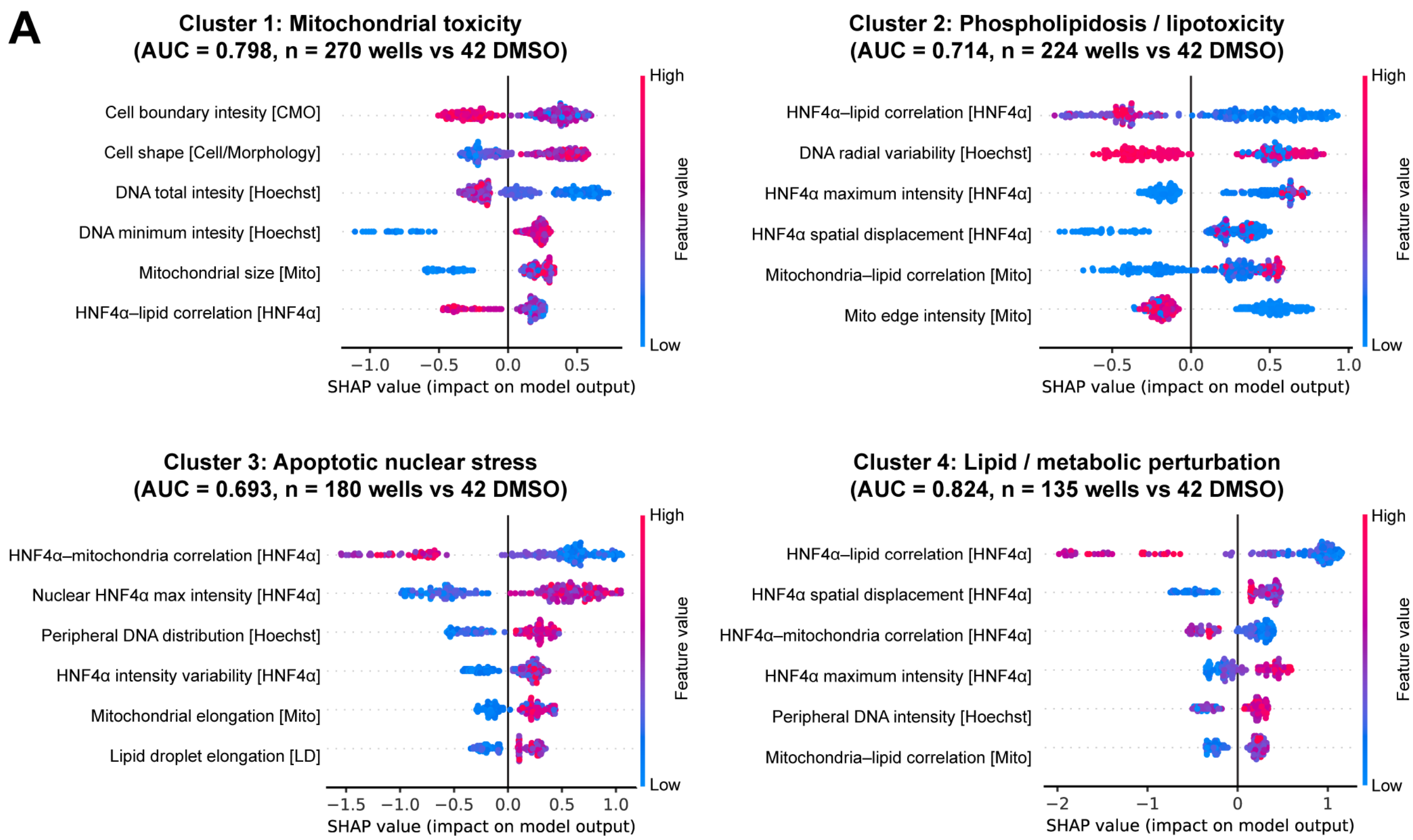

Supplemental Figure S3. SHAP-based interpretation of mechanistic hepatotoxicity clusters identified from HLO morphological profiling. SHAP (SHapley Additive exPlanations) summary plots showing the top contributing morphological features driving classification of each phenotypic cluster identified from line 64B HLOs. Each point represents an individual well, colored by normalized feature value (blue = low, pink = high). Positive SHAP values indicate features contributing toward cluster assignment. Cluster 1 was characterized primarily by mitochondrial morphology and DNA intensity features, consistent with mitochondrial toxicity. Cluster 2 showed strong contributions from lipid-, membrane-, and HNF4α-associated features, consistent with phospholipidosis/lipotoxicity. Cluster 3 was enriched for nuclear organization and chromatin-associated features, consistent with apoptotic or nuclear stress phenotypes. Cluster 4 was defined by subtler HNF4α-, lipid-, and mitochondria-associated spatial relationship features, consistent with delayed-onset metabolic idiosyncratic toxicity. AUC values and the number of cluster-associated wells used for model training are shown for each classifier.

bioRxiv preprint doi: https://doi.org/10.64898/2026.09.29.755355; this version posted October 4, 2026. The copyright holder for this preprint (which was not certified by peer review) is the author/funder, who has granted bioRxiv a license to display the preprint in perpetuity. It is made available under a CC-BY 4.0 International license.

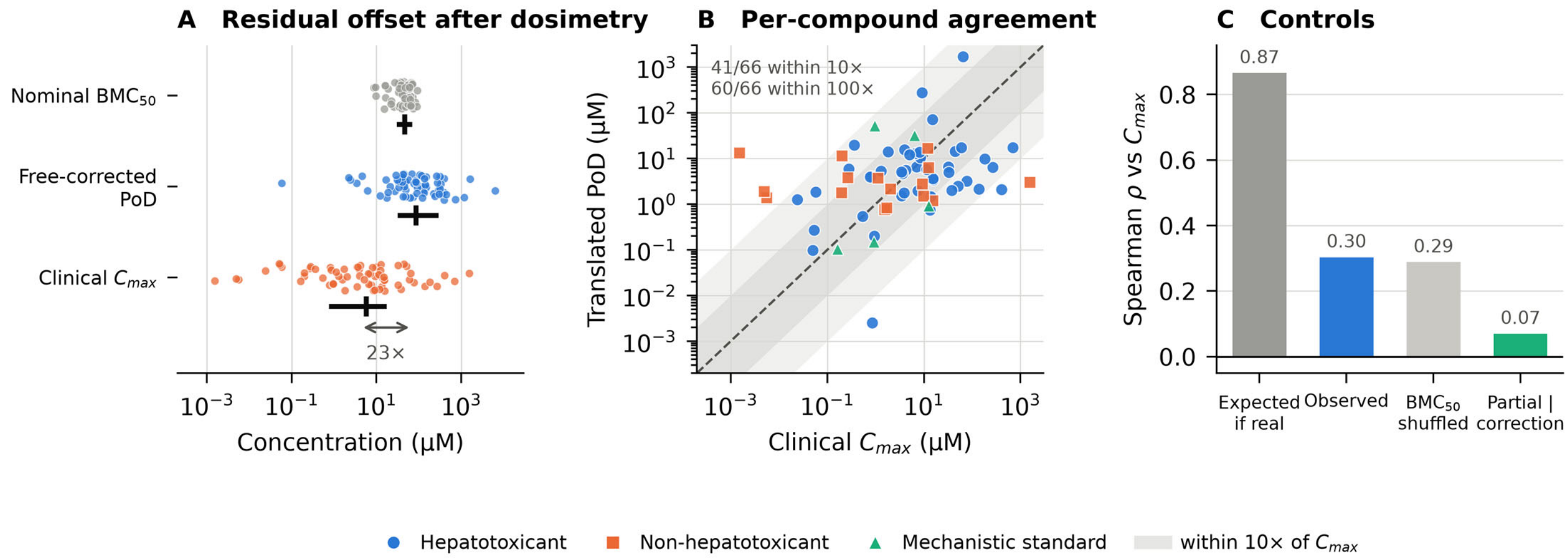

Supplemental Figure S4. Organoid points of departure relative to clinical exposure. (A) Distributions of nominal BMC_{50}, the free concentration after in-well dosimetry, and clinical total C_{max} for the 66 compounds with both a curated C_{max} and plasma free fraction. Points are individual compounds; bars mark median and interquartile range. The residual offset between the free-corrected point of departure and clinical exposure is 23-fold (95% CI 12–45). **(B)** Translated point of departure against clinical C_{max} for each compound. Dashed line, identity; shaded bands, within 10-fold and 100-fold of C_{max}. **(C)** Control analysis. The observed rank correlation is reproduced when BMC_{50} is permuted with the physicochemical correction retained, and vanishes when conditioned on that correction, whereas an assay with genuine compound-specific translation would be expected to give ρ = 0.87 under the same concentration range, censoring and inter-donor variability.

bioRxiv preprint doi: https://doi.org/10.64898/2026.09.29.755355; this version posted October 4, 2026. The copyright holder for this preprint (which was not certified by peer review) is the author/funder, who has granted bioRxiv a license to display the preprint in perpetuity. It is made available under a CC-BY 4.0 International license.

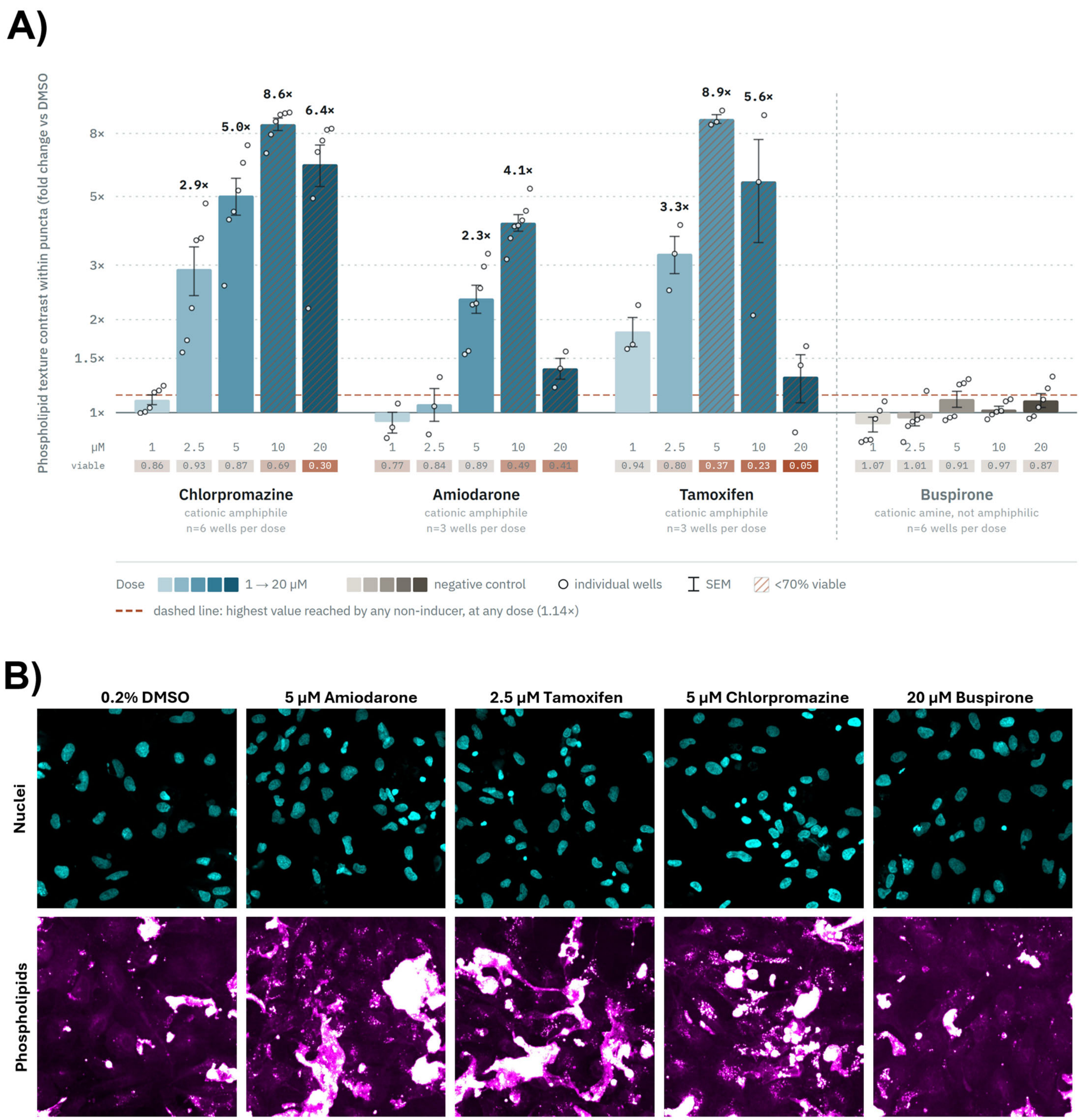

Supplemental Figure S5. Phospholipid-specific staining confirms phospholipidosis in 92F HLOs treated with cationic amphiphilic drugs. **(A)** Phospholipid puncta texture contrast after 72 h exposure, expressed as fold change over same-plate 0.2% DMSO ($\log_2$ axis). Chlorpromazine, amiodarone, and tamoxifen each increased dose-dependently, reaching 5.04×, 2.34×, and 3.27× while nuclei counts remained at 87%, 89%, and 80% of vehicle, respectively. Buspirone, a lysosomotropic cation that does not induce phospholipidosis, remained flat across the full dose range (0.91–1.10×), and no non-inducer exceeded 1.14× at any dose (dashed line). Bars, mean ± SEM of replicate wells; circles, individual wells. Striped bars indicate doses with fewer than 70% of vehicle nuclei; the strip below the axis gives the viable fraction for each bar. **(B)** Representative images of vehicle- and compound-treated cells. Nuclei, cyan; phospholipids, magenta hot LUT.
